# Reply to Wakolbinger-Habel, R.; Muschitz, C. Comment on “Kaufman et al. Popular Dietary Trends’ Impact on Athletic Performance: A Critical Analysis Review. *Nutrients* 2023, *15*, 3511”

**DOI:** 10.3390/nu15224710

**Published:** 2023-11-07

**Authors:** Matthew Kaufman, Levi Frehlich, Michael Fredericson

**Affiliations:** 1 Department of Orthopaedic Surgery, Stanford University, Redwood City, CA 94063, USA; mfred2@stanford.edu; 2 Stanford Lifestyle Medicine, Stanford University, Redwood City, CA 94063, USA; levi.frehlich@ucalgary.ca; 3 Cumming School of Medicine, University of Calgary, 3330 Hospital Drive NW, Calgary, AL T2N 4N1, Canada

We appreciate Wakolbinger-Habel and Muschitz’s comment [1]. The goal of our article, and particularly this section, is to highlight the potential pitfalls of adopting certain diets, including the vegan diet [2]. The goal was to warn those without proper monitoring who are adopting this diet that they have the potential for decreased bone mass density compared to omnivores, which has been supported in recent systematic reviews [3]. It seems through the study conducted by Wakolbinger-Habel et al. [4] that there is a possibility that this risk can be mitigated by a proper resistance training regimen. We do agree that more specificity could have been warranted in our manuscript; however, when looking at Figure 2 from Wakolbinger-Habel and Muschitz (2022) [4], it is noted that resistance-trained vegans are consistently lower than resistance-trained omnivores, and further, for tibial trabecular thickness, resistance-trained vegans have a lower microarchitecture than non-resistance-trained omnivores [4]. As we state within our review, this finding has not been replicated and represents a frontier of research that should be further investigated. Although this added specificity may have helped with clarity, we believe that our conclusions still accurately reflect the current state of the literature.
